# Present and Future of Dyslipidaemia Treatment—A Review

**DOI:** 10.3390/jcm12185839

**Published:** 2023-09-08

**Authors:** Iveta Merćep, Andro Vujević, Dominik Strikić, Ivana Radman, Ivan Pećin, Željko Reiner

**Affiliations:** 1 Department of Internal Medicine, School of Medicine, University of Zagreb, 10000 Zagreb, Croatia; imercep@gmail.com (I.M.); ipecin@kbc-zagreb.hr (I.P.); 2 Division of Clinical Pharmacology, Department of Internal Medicine, University Hospital Centre Zagreb, 10000 Zagreb, Croatia; avujevi@kbc-zagreb.hr; 3 Department of Ophthalmology, Sestre Milosrdnice University Hospital Centre, 10000 Zagreb, Croatia; ivana.radman@gmail.com; 4 Division of Metabolic Diseases, Department of Internal Medicine, University Hospital Centre Zagreb, 10000 Zagreb, Croatia; zreiner@kbc-zagreb.hr; 5 Department of Cardiology and Congenital Diseases of Adults, Polish Mother’s Memorial Hospital Research Institute, 93-338 Lodz, Poland

**Keywords:** dyslipidaemia, cardiovascular disease, major cardiovascular events (MACEs), atherosclerosis, new lipid lowering drugs

## Abstract

One of the greatest burdens on the healthcare systems of modern civilization is cardiovascular diseases (CVDs). Therefore, the medical community is looking for ways to reduce the incidence of CVDs. Simple lifestyle changes from an unhealthy to a healthy lifestyle are the cornerstone of prevention, but other risk factors for cardiovascular disease are also being currently targeted, most notably dyslipidaemia. It is well known that lowering serum lipid levels, and in particular lowering elevated LDL-cholesterol, leads to a reduction in major cardiovascular events. Although the focus to date has been on LDL-cholesterol levels and lowering them with statin therapy, this is often not enough because of increased concentrations of other lipoprotein particles in the serum and residual cardiovascular risk. Since lowering LDL-cholesterol levels is successful in most cases, there has been a recent focus on lowering residual cardiovascular risk. In recent years, new therapeutic options have emerged that target triglyceride-rich lipoproteins, lipoprotein (a) and apolipoproteins C and B. The effects of these drugs on serious adverse cardiovascular events are not yet known, but recent studies with some of these drugs have shown significant results in lowering total lipid levels. The aim of this review is to present the current therapeutic options for the treatment of dyslipidaemia and to describe the newly approved drugs as well as the drugs that are still in development. Although at this stage we cannot say with certainty whether these agents will be approved and widely used, it is safe to say that our views on the treatment of dyslipidaemia are certainly changing.

## 1. Introduction

Cardiovascular disease (CVD) is the leading cause of death worldwide, claiming an estimated 17.9 million lives each year [1]. This heterogeneous group of diseases includes coronary heart disease, cerebrovascular disease (especially strokes), peripheral arterial disease and a variety of other conditions [2]. The vast majority of risk factors for cardiovascular disease are modifiable, such as diabetes mellitus, smoking, hypertension, obesity, dyslipidaemia and physical inactivity [2]. Dyslipidaemia, defined as elevated plasma concentrations of total cholesterol, LDL-cholesterol, triglycerides and decreased HDL-cholesterol, or a combination of these characteristics, is one of the most important modifiable risk factors for CVDs [3]. In 2019, approximately 44% of ischaemic heart disease deaths and 22% of ischaemic stroke deaths could be attributed to elevated plasma LDL-cholesterol levels [3]. Modifiable risk factors for dyslipidaemia are similar to those associated with CVDs and include diabetes, obesity, physical inactivity and a high intake of saturated or trans fats [4]. However, some dyslipidaemias are inherited diseases, of which familial hypercholesterolaemia is the most important and common. Although changes in lifestyle and dietary habits and increased use of lipid-lowering drugs have led to a decrease in the prevalence of dyslipidaemia in industrialised countries, the prevalence is still high [5]. Data for the US show that in 2015 to 2018, 38.1% of adults had total cholesterol ≥200 mg/dL (~5.2 mmol/L), 27.8% of adults had LDL-cholesterol ≥130 mg/dL (~3.4 mmol/L) in, 21.1% had triglycerides ≥150 mg/dL (~1.7 mmol/L), and 17.2% had HDL-cholesterol < 40 mg/dL (~1.0 mmol/L) [6]. Treatment with statins for people who need therapy increased in 2013–2016 compared to 2001–2004, but there is still room for improvement—especially among women [6]. Significant decreases in total and non-HDL-cholesterol have been observed in developed European countries and high-income North American countries [3]. These data are followed by a significant decline in age-standardised death rates (ASDR) for ischaemic heart disease attributable to high LDL-cholesterol levels per 100,000 of the general population in Europe and high-income North American countries [3]. The decline in age-standardised death rate (ASDR) from 1990 to 2019 ranged from 143.38 (181.55–107.26) to 71.86 (95.36–49.06) in Central Europe, from 78.21 (98.64–58.37) to 27.80 (36.90–19.53) in Western Europe, and from 97.91 (120.57–74.74) to 34.35 (45.26–24.82) in high-income North American countries [3]. Even though the situation in Central Europe has improved significantly, there are still countries like Czechia with worrying data. In the European Health Examination Survey (EHES) published in 2014, dyslipidaemia was found in 77% of men and 62% of women [7]. This was associated with a low prevalence of treatment with lipid-lowering drugs (30% in men and 23% in women) and a low success rate in controlling plasma lipid levels (19% in men and 52% in women) [7]. The increasing but still inadequate use of lipid-lowering drugs in most countries can be explained by the substantial decrease in costs, especially for statins, which are now available as generics, and by changes in guidelines recommending earlier initiation of treatment with lipid-lowering drugs [3]. In developing countries, lipid-lowering drug use and lifestyle and dietary changes are not improving as rapidly as in high-income countries. In low-income countries, awareness of dyslipidaemia, and therefore its diagnosis and treatment, is unfortunately not at the desired level, resulting in a slower decline in deaths associated with dyslipidaemia [3]. Regions such as the Middle East and North Africa, therefore, show less progress in ASDR for ischaemic heart disease attributable to high plasma LDL-cholesterol levels over the period of 1990–2019 (from 127.73 (161.60–96.20) to 91.11 (118.68–66.49)) [3].

Dyslipidaemias can be primary (inherited diseases such as familial hypercholesterolaemia) and secondary. Secondary dyslipidaemias are associated with other conditions such as uncontrolled diabetes mellitus, obesity, physical inactivity, certain medications or underlying physical diseases [8,9]. Diseases associated with dyslipidaemia include nephrotic syndrome, obstructive jaundice, Cushing’s syndrome, excessive alcohol consumption and others [10]. A breakthrough advance in the treatment of dyslipidaemia is the strategic targeting of lipoprotein (a), a unique lipoprotein particle consisting of an LDL-cholesterol-like core and an additional apolipoprotein called apolipoprotein(a) [apo(a)]. It has structural similarity to LDL-cholesterol, which allows it to be incorporated into arterial plaques, promoting the development of atherosclerotic lesions while impairing the breakdown of blood clots [11]. Recent research highlights that elevated Lp(a) concentrations are associated with a gradual increase in the likelihood of myocardial infarction, thus increasing the overall risk of cardiovascular disease. As a result, the therapeutic focus in the treatment of dyslipidaemia has evolved beyond the exclusive lowering of LDL-cholesterol. Instead, attention has shifted to interventions that have the dual goal of lowering both LDL-cholesterol and Lp(a) levels. This multifaceted approach recognises the intricate interplay between lipoprotein(a) and cardiovascular health and calls for a nuanced recalibration of treatment strategies [11,12].

Currently, cardiovascular disease (CVD) risk assessment relies mainly on the use of the SCORE (Systemic Coronary Risk Estimation) system. Although effective, the evolution of risk assessment has been underlined by the recent introduction of the SCORE-2 and SCORE-OP systems [13]. This progressive refinement reflects the quest for accuracy in CVD risk assessment. The basic principle of risk assessment is that a higher risk requires more robust intervention strategies. Conversely, the concept of residual CVD risk introduces another dimension—a risk that persists despite the use of standard medical therapies and interventions aimed at attenuating conventional cardiovascular risk factors [14]. While clinical guidelines provide clarity on cardiovascular risk reduction, the concept of residual cardiovascular risk remains a compelling consideration that deserves attention from the medical community. In the dynamic landscape of cardiovascular health, the discourse of residual CVD risk needs to be further explored and understood. The aim of this review article is to highlight novel therapeutic approaches in the treatment of dyslipidaemia. The focus is primarily on approved drugs, but also on new agents currently in the pipeline.

## 2. Methods

For this review, PubMed, Scopus, Embase and Web of Science were searched from October 2022 to June 2023 using the keywords dyslipidaemia, new therapeutic approaches, serious cardiovascular events (MACEs), atherosclerosis, new lipid-lowering drugs and statins. 

## 3. Present Therapeutic Options

### 3.1. Statins

Statins are the most commonly prescribed lipid-lowering drugs. The global statin market reached USD 14.3 billion in 2021 and is expected to reach more than USD 17 billion by 2027 [15]. Statins approved by the European Medicines Agency (EMA) and the US Food and Drug Administration (FDA) include atorvastatin, simvastatin, rosuvastatin, fluvastatin, pitavastatin, lovastatin and pravastatin. They are used for primary and secondary prevention of cardiovascular disease. Statins are competitive 3-hydroxy-3-methylglutaryl-coenzyme A (HMG-CoA) reductase inhibitors. HMG-CoA reductase is a rate-limiting enzyme in cholesterol biosynthesis [13]. By inhibiting the enzyme, they reduce intracellular cholesterol synthesis, thereby decreasing intracellular cholesterol levels and increasing low-density lipoprotein receptor (LDLR) expression, thus removing more cholesterol-rich LDL particles from the circulation [13]. Statins are effective in lowering LDL-cholesterol levels and the associated reduction in the risk of major cardiovascular events (MACEs) [16]. The adjusted hazard ratio for MACEs is correlated with achieved LDL-cholesterol levels [16]. However, there is still a significant percentage of patients who do not achieve the targeted LDL-cholesterol levels with statin monotherapy [16]. Statin use is associated with a moderate increase in HDL-C levels and a decrease in triglyceride (TG)levels, with simvastatin and rosuvastatin achieving better results than atorvastatin for these lipids and lipoproteins [17]. The best relative increase in HDL-C levels is obtained in patients with low HDL-C levels and/or high TG levels at baseline [17]. Better results in HDL-C and TG levels are obtained when fibrate or niacin is added to statin therapy [18]. It is important to note that statins have little to no effect on Lp(a) levels and that elevated Lp(a) is a risk factor even in patients with low LDL-cholesterol levels [19,20]. Statins are also associated with the conversion of atherosclerotic plaques into high-calcium-density lesions, which are associated with a lower cardiovascular risk [21]. Commonly reported adverse effects of statin therapy include muscle pain, liver damage and insulin resistance, while prolonged treatment with the highest statin doses has been associated with an increase in diabetes rates [22]. A link between statin treatment and neoplastic disease, dementia and cognitive impairment has been refuted [23,24]. Long-term statin treatment promotes insulin resistance and may lead to the need for insulin treatment sooner [25]. All these adverse effects, together with a lack of awareness of the health risks associated with dyslipidaemia, result in poor adherence to statin treatment, reported to be 49% or even lower [26]. An innovative approach to statin treatment is the polypill strategy, also known as a fixed-dose combination (FDC). This method combines multiple drugs into a single pill to streamline dosing schedules and improve patient adherence to treatment protocols. In practise, it has been shown that patients more consistently take statins when they are combined with antihypertensives or antiplatelets in a single medication [27,28]. However, it is worth noting that even when the polypill strategy is implemented strategically, the problem of statin adherence remains, especially in primary prevention. This paradoxical association between low statin compliance and increased cardiovascular disease (CVD) mortality underscores the importance of further improving strategies to promote patient participation and adherence [29]. The reduction in cardiovascular risk achieved with statin treatment outweighs the risk of adverse effects [22]. Therefore, statins remain the first choice for the treatment of dyslipidaemia, especially hypercholesterolaemia. However, in recent years and in the future, the treatment of dyslipidaemia will tend to shift from statin monotherapy to FDC therapy. These new treatment approaches involve a combination of novel dyslipidaemia drugs with statin drugs. 

### 3.2. Fibrates

Fibrates are old lipid-lowering agents that are no longer used as frequently as statins. They are agonists of the peroxisome proliferator-activated receptor (PPAR), particularly for PPARα [30]. Activation of PPARα leads to a cascade of effects associated with a decrease in triglyceride levels and an increase in HDL-cholesterol levels [30]. These effects include induction of lipoprotein lipase (LPL), suppression of apolipoprotein C- II and CIII expression, increase in gene expression of apolipoprotein A-I, and induction of acyl-coenzyme A synthetase—an enzyme critical for lipid uptake and intracellular lipid metabolism [31,32,33]. Fibrates also have an anti-atherogenic effect due to the inhibition of vascular cell adhesion molecules (VCAMs) [34]. However, fibrates are primarily drugs for lowering elevated triglycerides rather than elevated LDL-cholesterol levels. Although generally well tolerated, fibrates have some well-described adverse effects which are the reason for the substantial decline in fibrate prescription and adherence to fibrate therapy. In addition to muscle and liver toxicity similar to that of statins, adverse effects include gastrointestinal discomfort and exacerbation of co-existing gastrointestinal conditions, skin reactions, peripheral neuropathy, erectile dysfunction and lithogenicity due to stimulation of biliary cholesterol secretion [35]. The effect of fibrates on the primary prevention of cardiovascular disease is much less than the effect of statins [36]. Consequently, statins hold the major share of the lipid-lowering market. However, as there is a residual cardiovascular risk caused mainly by high triglyceride and low HDL-cholesterol levels, the addition of fibrates to statin monotherapy may benefit patients at residual risk [30,37,38]. Recent studies in animal models have linked pemafibrate to a reduction in the risk of abdominal aortic aneurysm rupture, but further studies are needed to confirm this [39]. Although great expectations have been placed on pemafibrate, a selective peroxisome proliferator-activated receptor alpha modulator (SPPARMα) [39,40], the PROMINENT trial of this drug failed to show a reduction in the incidence of cardiovascular events in patients with type 2 diabetes and dyslipidaemia, although pemafibrate did lower triglycerides, VLDL-cholesterol, residual cholesterol and apolipoprotein C III [41].

### 3.3. Bempedoic Acid

**Figure 1:**
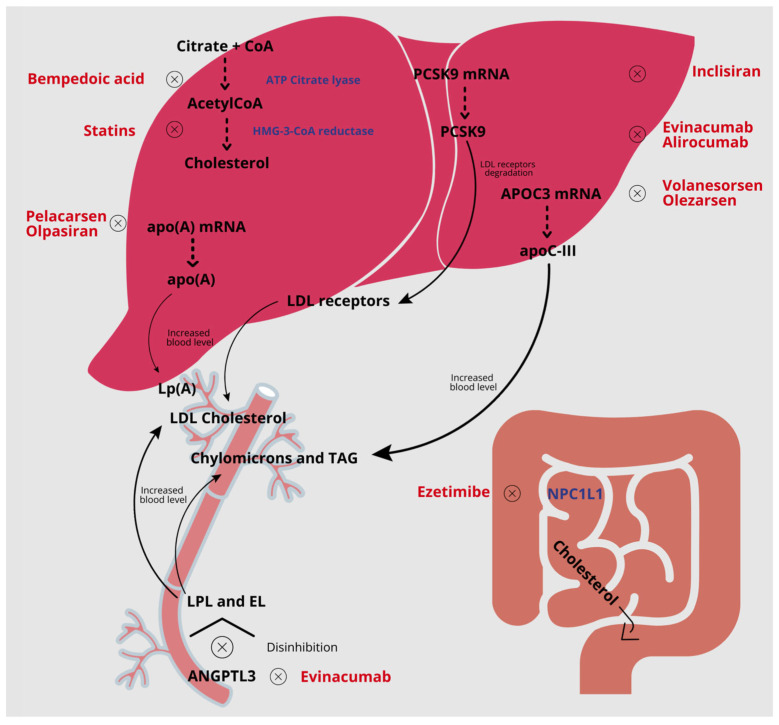
Schematic representation of the mechanism of action of lipid-lowering agents.

Bempedoic acid (BPA) is a prodrug activated by very long-chain acyl-CoA synthetase, an enzyme found in liver and kidney but not in muscle cells. The active metabolite of bempedoic acid inhibits ATP citrate lyase, the enzyme upstream of HMG-CoA reductase in the de novo cholesterol synthesis pathway [42]. It is very well absorbed, has good gastrointestinal tolerance and good bioavailability, and is administered once daily in the form of a 180 mg pill [43]. Similar to the pharmacodynamics of statins, the reduction in cholesterol synthesis leads to an upregulation of LDL-cholesterol receptor expression [42]. Recent data suggest that there are other mechanisms of the lipid-lowering effect of bempedoic acid. These mechanisms include the reduction of ketohexokinase in the liver, resulting in lower fructose uptake and reduced lipogenesis, increased expression of patatin-like phospholipase domain-containing protein 3 (PNPLA 3), which has been associated with a lower incidence of non-alcoholic fatty liver disease (NAFLD), and PPARα agonist activity similar to fibrates [44] [Figure 1]. The efficacy and safety of BDA has been investigated in five studies: CLEAR Harmony, Wisdom, Serenity, Tranquilly and Outcomes [42]. CLEAR Harmony (Assessment of the Long-Term Safety and Efficacy of Bempedoic Acid) and CLEAR Wisdom (Effect of Bempedoic Acid vs. Placebo Added to Maximally Tolerated Statins on Low-Density Lipoprotein Cholesterol in Patients at High Risk for Cardiovascular Disease) examined the LDL-cholesterol reduction of bempedoic acid compared to placebo in patients receiving maximally tolerated statin therapy [45,46]. Both studies showed a significant reduction in LDL-cholesterol levels compared with placebo (−17.4% and −18.1%) at week 12 of therapy [45,46]. Statistically significant, although slightly lower than that at week 12, reductions in LDL-cholesterol levels were still seen at week 52 of therapy [45,46]. In addition to the reduction in LDL-cholesterol, a decrease in non-HDL-cholesterol, total cholesterol, apolipoprotein B and high-sensitivity C-reactive protein was seen in the bempedoic acid group in both trials [45,46]. The reported frequency of adverse events was similar in the bempedoic acid and placebo groups. The only reported adverse effect was an increased incidence of gout and urinary tract infections in the bempedoic acid group [45,46]. CLEAR Tranquillity (Evaluation of the Efficacy and Safety of Bempedoic Acid as Add-on to Ezetimibe Therapy in Patients with Elevated LDL-Cholesterol) investigated the effect of bempedoic acid compared to placebo in patients already receiving ezetimibe therapy. At week 12, a statistically significant reduction in LDL-cholesterol (−28% compared to placebo) was observed, as well as reductions in non-HDL-cholesterol, total cholesterol, apolipoprotein B and high-sensitivity C-reactive protein [47]. Adverse effects in the Tranquillity trial were similar in the bempedoic acid and placebo groups, demonstrating that bempedoic acid is well tolerated. The CLEAR Serenity trial investigated the lowering of LDL-cholesterol levels by bempedoic acid in patients who could not tolerate high-dose statin therapy. The results were comparable to the previously mentioned CLEAR studies with a −21.4% reduction in LDL-cholesterol compared to placebo at week 12, along with non-HDL-C, high-sensitivity C-reactive protein, total cholesterol and apolipoprotein B [48]. An important observation in the Serenity trial was the weaker effect of LDL-cholesterol lowering in patients with diabetes mellitus [49]. CLEAR Outcomes was a large study aimed at analysing the efficacy of bempedoic acid on cardiovascular outcomes. The results of this study, which were published recently, showed that treatment with bempedoic acid was associated with a reduction in the number of strokes or coronary revascularisations in a subgroup of high-risk patients in primary prevention, but had no significant effects. Adverse effects of bempedoic acid included a higher incidence of gout, cholelithiasis and increases in serum creatinine, uric acid and liver enzyme levels [43,50]. The same study suggests that in patients intolerant of statins, treatment with bempedoic acid was associated with a lower risk of MACEs [50,51,52]. A meta-analysis associated treatment with bempedoic acid with a reduction in cardiovascular risk, new-onset diabetes and worsening diabetes [51,53]. Bempedoic acid could therefore be a useful adjunct in the treatment of lipid-lowering agents. Based on the results of all these studies, a position paper was recently published recommending the use of bempedoic acid in atherosclerotic CVD, familial hypercholesterolaemia and statin intolerance. The authors of this paper also points out that while there is not yet sufficient data to support the role of bempedoic acid in the primary prevention of CVD, its beneficial effects on plasma glucose and inflammatory markers suggest that this drug may be a rational choice in patient-centred care for certain primary prevention groups [50,51,54]. Currently, bempedoic acid is used as either a monotherapy in patients with well-documented statin intolerance or as a part of FDC medication together with statins or other lipid-lowering medications. 

### 3.4. Ezetimibe

Ezetimibe is a selective inhibitor of cholesterol absorption. It is an antagonist of the Niemann-Pick C1-Like 1 (NPC1L1) protein on intestinal epithelial cells [55]. Inhibition of the NPC1L1 protein causes a reduction in cholesterol absorption and subsequently a reduction in cholesterol levels in the liver, leading to better excretion of cholesterol from the blood [55]. Ezetimibe is available in the form of 10 mg tablets. It can be used as a monotherapy, but is incomparably more commonly used in combination with statins, fibrates or bempedoic acid. There are already fixed combinations of ezetimibe with statins and bempedoic acid on the market that have been approved by the FDA and the EMA. Ezetimibe has good oral bioavailability and is better absorbed in older patients [55]. This difference between older and younger people does not require dose adjustment. Although not commonly used, ezetimibe monotherapy is associated with statistically significant reductions in LDL-cholesterol (−18.58%), total cholesterol (−13.46%), TGs (−8.06%) and increases in HDL-cholesterol (+3%) [56]. Ezetimibe monotherapy is well tolerated with few adverse effects, making it a good option for patients who cannot tolerate statins [56]. Ezetimibe therapy in combination with various statins results in greater reductions in LDL-cholesterol and TGs and increases in HDL-cholesterol compared to statin monotherapy with a similar side effect profile [57,58,59]. It is important to note that the addition of ezetimibe is not associated with a reduction in inflammation of atherosclerotic plaques [60]. The EFECTL study showed similar results for the addition of ezetimibe to fibrate therapy [61]. The study IMPROVE-IT investigated the effects of adding ezetimibe to statin therapy on reducing stroke and adverse cardiovascular outcomes. Ezetimibe was associated with a reduction in adverse cardiovascular outcomes, particularly in high-risk patients and patients with diabetes mellitus, and a reduction in secondary strokes [62,63]. In the primary prevention of stroke, ezetimibe therapy did not achieve better results than statin monotherapy [62]. The study SEAS investigated the use of ezetimibe in the prevention of adverse events related to the aortic valve in patients with aortic valve stenosis. The risk of adverse events related to the aortic valve was not reduced by ezetimibe therapy, but ezetimibe treatment reduced adverse cardiovascular outcomes overall [64]. The SEAS study showed a higher incidence of cancer in the ezetimibe group, but similar results were not reported by any other study [64]. High-dose statins or a combination of statins with ezetimibe have also been shown to reduce the number of atherogenic oxidised LDL particles more than low-to-moderate intensity statin therapy alone [65]. The addition of ezetimibe has significant potential to reduce residual cardiovascular risk in patients who do not achieve target LDL-cholesterol levels on standard statin therapy.

### 3.5. PCSK-9 Inhibitors

Proprotein convertase subtilisin/kexin type 9 (PCSK9) is a glycoprotein secreted mainly by the liver. It binds to LDLR on the cell surface and stimulates endocytosis and lysosomal degradation of LDLR [66]. PCSK9 is also involved in the secretion of VLDL and Apo B and in promoting endothelial inflammatory processes [66]. The degradation of LDLR attenuates the endocytosis of LDL particles and leads to higher LDL-cholesterol levels in the blood. PCSK9 inhibitors are relatively new drugs that inhibit PCSK9, reduce LDLR degradation and lower serum LDL-cholesterol by increasing LDL particle uptake. There are currently two approved monoclonal antibodies that inhibit PCSK9: alirocumab and evolocumab. The third agent studied was bococizumab, but the trials were stopped due to increased immunogenicity (causing inconsistent LDL-cholesterol lowering) and injection site reactions [67]. The effect of evolocumab has been investigated in numerous studies in patients with dyslipidaemia, heterozygous familial hypercholesterolaemia (HeFH) and homozygous familial hypercholesterolaemia (HoFH). The most important studies were the FOURIER, DESCARTES, OSLER and TESLA studies. These trials associated treatment with evolocumab with a significant reduction in LDL-cholesterol levels, which averaged 50–60% in patients without HoFH [68]. Patients with HoFH have very low residual LDLR activity. Conventional lipid-lowering therapy with statins has little effect on lowering LDL-cholesterol in these patients. The study TESLA has shown that therapy with evolocumab also leads to a reduction in LDL-cholesterol in patients with HoFH, although the reduction is lower (−30%) due to the lack of LDLR activity [69]. The studies mentioned earlier have also shown that treatment with evolocumab leads to reductions in total cholesterol, non-HDL-cholesterol, apo B and, most importantly, Lp(a)–lipoprotein, which is associated with a residual risk of cardiovascular disease and on which older lipid-lowering agents, including statins, have little or no effect [68]. Significant improvement to the lipid profile of patients with dyslipidaemia treated with evolocumab in addition to their maximally tolerated statin therapy is associated with a reduction in cardiovascular risk [70]. In high-risk patients, the addition of evolocumab to statin + ezetimibe therapy was associated with an almost 3-fold reduction in MACEs and a significant reduction in Lp(a) (−38.84 ± 32.40%) [71]. The open-label study EVOCATION investigated the effect of evolocumab and microvascular stability in patients undergoing percutaneous coronary intervention (PCI), but pretreatment with evolocumab did not prevent microvascular dysfunction [72]. The effect of alirocumab has also been investigated in many studies, the largest of which is the ODYSSEY LONG TERM study. The ODYSSEY LONG TERM study showed a 61% reduction in LDL-cholesterol at week 24 and a long-term reduction of −52.4% at week 72, along with an overall improvement in lipid profile including Lp(a) [73]. Additional analyses of the results from ODYSSEY and the results of the ODYSSEY OUTCOMES study showed that treatment with alirocumab was associated with a significant reduction in MACEs [74,75,76]. Alirocumab also reduced the risk of stroke, independent of baseline LDL-cholesterol and history of cerebrovascular disease [76]. The non-lipidic effects of alirocumab are also beneficial, contributing to a reduction in the risk of MACEs. Inflammation of the vessel walls is one of the major pathophysiological steps in plaque rupture and thrombosis. Clinical studies showed a reduction in oxidative stress, inflammatory cytokines such as IL-18, IL-6 and TNF-α and a reduction in metalloproteinase 2 (MMP-2), osteopontin (OPN) and osteoprotegerin (OPG), key factors in vessel wall inflammation, in patients treated with alirocumab [77,78,79]. Alirocumab has recently been studied in patients on stable dialysis treatment and has been shown to be effective and safe in these patients [80]. Both evolocumab and alirocumab are approved by the EMA and FDA. The usual administration regimen for alirocumab is 75 mg or 150 mg subcutaneously every 2 weeks or 300 mg subcutaneously every 4 weeks. Evolocumab is administered subcutaneously at a dose of 140 mg every 2 weeks or 420 mg once a month. Both drugs are approved for use in combination with statins and/or other lipid-lowering drugs or as a monotherapy in patients who cannot tolerate statin therapy. PCSK9 inhibitors have a good safety profile. The only adverse effect that could be directly associated with PCSK9 inhibitors is a reduction in vitamin E levels [81]. Intensive lowering of LDL-cholesterol could interfere with vitamin E transport and explain lower vitamin E levels. The biggest obstacle to wider use of PCSK9 inhibitors at present is their cost. There are reports calling for a price reduction of more than 37% or tighter prescription restrictions so that PCSK9 inhibitors could be cost-effective [82].

### 3.6. Inclisiran

Inclisiran is a double-stranded small interfering RNA (siRNA) that targets PCSK9 mRNA and inhibits hepatic PCSK9 synthesis [83]. The liver specificity of inclisiran is provided by conjugation with triantennary N-acetylgalactosamine carbohydrate (GalNAC), which facilitates uptake by the asialoglycoprotein receptor, which is highly expressed in hepatocytes but not in muscle [83]. The effect of reduced PCSK9 expression leads to results similar to those from inhibition of PCSK9 with monoclonal antibodies. The efficacy of inclisiran has been most thoroughly investigated in the ORION studies. The ORION Studies showed that treatment with inclisiran lowers LDL-cholesterol, with the best results (−50% reduction) achieved with two doses of 300 mg Inclisiran per year [84]. Although the reduction is somewhat less than with PCSK9 monoclonal antibodies, inclisiran’s administration schedule, together with its long-lasting effect, is an advantage of inclisiran. The reduction in LDL-cholesterol was significant throughout the 240 days of the study [84]. Similar to PCSK9 inhibitors, treatment with inclisiran was also associated with a decrease in TGs, Apo B, Lp(a) and total cholesterol and an increase in HDL-C [84]. There are currently a number of studies analysing the risk of possible adverse effects as well as the cardiovascular outcomes of inclisiran treatment. Reports on MACE risk reduction in patients treated with inclisiran are expected in the coming years, namely ORION-4 and VICTORION-2 PREVENT, but initial publications suggest overall favourable results in MACE risk reduction [85,86]. Inclisiran is generally well tolerated and the only commonly reported side effect is injection site reactions, which are usually mild [87,88]. Inclisiran is approved by the EMA and the FDA. It is administered as a subcutaneous injection at a dose of 284 mg. After the first injection, the second dose is administered after 3 months and the drug is continued every 6 months. A major advantage of this administration regimen is the high percentage of treatment adherence and patient compliance [89] [Table 1].

## 4. Therapeutic Options beyond Statins, Bempedoic Acid, Ezetimibe and PCSK9 Inhibition

### 4.1. ANGPTL3 Inhibitors

Angiopoietin-like protein 3 is a polypeptide that plays an important role in lipid metabolism. It is mainly secreted in the liver. ANGPTL3 acts as an inhibitor of lipoprotein lipase (LPL) and endothelial lipase (EL), thereby increasing circulating levels of triglycerides and LDL-cholesterol [90]. Inhibition of ANGPTL3 leads to disinhibition of LPL and EL and thus to a decrease in triglycerides and LDL-cholesterol [91]. Inhibition of ANGPTL3 by monoclonal antibodies and antisense oligonucleotides has been the focus of recent clinical trials. Evinacumab, a monoclonal antibody against ANGPTL3, has been studied in patients with HoFH. The reduction in LDL-cholesterol induced by evinacumab is statistically significant, with reductions of −49 and −59% reported [92,93]. In patients with mixed dyslipidaemia, evinacumab has a rapid effect on lowering triglycerides, which decreased by 77 and 83% on days 2 and 3, respectively [94]. Commonly reported adverse effects included flu-like symptoms and mild increases in aminotransferases and creatine kinase [92,94]. No serious adverse effects were reported in patients treated with evinacumab. Vupanorsen is an antisense oligonucleotide that decreases ANGPTL3 expression. It has shown good results with significant reductions in non-HDL-C, apoB, total cholesterol and especially TGs [95,96,97]. Vupanorsen has also increased insulin sensitivity and lowered fasting blood glucose in diabetics [97]. However, although vupanorsen produced a significant improvement in the lipid profile in treated patients, it was withdrawn from the clinical development programme [98]. The clinical trial TRANSLATE-TIMI was discontinued due to hepatic steatosis and >3-fold increases in alanine and aspartate aminotransferases attributed to treatment with vupanorsen [95]. Evinacumab is currently approved by the EMA and FDA for the treatment of HoFH. It is administered as an intravenous infusion at the recommended dose of 15 mg/kg. Evinacumab and new ANGPTL3 inhibitors that may be produced in the future could be important drugs for patients with residual cardiovascular risk associated with high levels of TGs.

### 4.2. Drugs Targeting Lp(a)—Pelacarsen, Olpasiran and SLN360

The previously mentioned drugs are already approved and effective in lowering LDL-cholesterol, total cholesterol and/or TGs. Although the lowering of other lipoproteins and lipids in the blood is usually accompanied by some reduction in Lp(a), high Lp(a) levels remain an important factor in cardiovascular risk, and to date there is no drug that significantly lowers Lp(a). Therefore, drugs are currently being developed that directly target Lp(a) production or its effects. Pelacarsen is an antisense oligonucleotide that binds to hepatocyte apo(a) mRNA and forms an ASO/mRNA complex that prevents translation of apolipoprotein(a) [9]. This leads to a decrease in Lp(a) production and a decrease in serum Lp(a) levels. Pelacarsen has already shown promising results in phase 2 clinical research. A dose-dependent reduction in Lp(a) and a mean percentage reduction of 35% at a dose of 20 mg every 4 weeks, 56% at 40 mg every 4 weeks, 58% at 20 mg every 2 weeks, 72% at 60 mg every 4 weeks and 80% at 20 mg every week have been reported [99]. In another phase 2 study, a dose-dependent 29% to 67% reduction in Lp(a) was reported along with a 2–19% reduction in LDL-cholesterol and a 3–16% reduction in apoB [100]. Pelacarsen is well tolerated, although adverse effects such as myalgias, urinary tract infections, injection site reactions and headaches have been reported [99]. A phase 3 trial is currently underway (HORIZON), investigating the effect of pelacarsen on MACEs, and is expected to be completed by 2025 [101]. Olpasiran is an siRNA that directly inhibits the translation of Lp(a) mRNA in hepatocytes. The reduction in translation leads to a decrease in serum Lp(a) levels. Phase 1 studies reported that Lp(a) levels were reduced by up to 90% after a single dose of 9 mg or more for more than 6 months, with no safety concerns [102]. Another siRNA that targets Lp(a) mRNA is SLN360. It was recently studied in a small single-dose phase 1 trial with 32 participants. The study showed dose-dependent Lp(a) reductions of −10%, −46%, −86%, −96% and −98% compared to the placebo group with −30 mg, −100 mg, −300 mg and −600 mg SLN360 groups, respectively [103]. There was a long period of time during which Lp(a) levels were reduced, suggesting that subcutaneous administration may also be effective when given more than once in 150 days [103]. 

### 4.3. Volanesorsen and Olezarsen

Another component of residual cardiovascular risk in patients who have reached the target level for LDL-cholesterol is the TG value. Drugs are currently being developed that could potentially reduce TGs and chylomicrons (TG-rich lipoproteins). Apolipoprotein C III is a small glycoprotein secreted by the liver and to a lesser extent the small intestine that binds to almost all lipoproteins—HDL, LDL and triglyceride-rich lipoproteins [104]. It inhibits LPL and hepatic uptake of triglyceride-rich lipoproteins (TRL), leading to an increase in TGs [104]. Volanesorsen is the first antisense oligonucleotide developed that targets apo-C III production [105]. Decreasing the production of Apo-C III decreases chylomicrons and thus TGs. Volanesorsen has been studied in phase 2 and 3 clinical trials. The most recent meta-analysis found that treatment with volanesorsen resulted in an average 74% decrease in TGs, a 72% decrease in VLDL, a 69% decrease in apo-B48, an 80% decrease in apo-CIII and a 46% increase in HDL-C [106]. The negative finding was an increase in LDL-cholesterol [106]. Volanesorsen has also been studied in patients with familial hyperchylomicronaemia syndrome (FCS). In the ReFOCUS study, treatment with this drug was associated with an improvement in physical symptoms and anxiety associated with the disease [107]. Considering that extreme hypertriglyceridaemia is a risk factor for acute pancreatitis, reducing TGs in patients with volanesorsen is important for reducing the incidence of pancreatitis [108]. The safety profile of volanesorsen is relatively good, but one must be aware of the potential adverse effects. The most commonly described effects are injection site reactions and upper respiratory tract infections compared to placebo in several studies [106,109]. The only serious adverse effects reported with volanesorsen treatment were thrombocytopenia and serum sickness [108,110]. The drug is administered subcutaneously at a dose of 285 mg. At the beginning of treatment, it is administered once a week. After 3 months, patients who have achieved a sufficient reduction in TGs can continue to receive the drug once every 2 weeks [111]. Due to the adverse effects, frequent checks of the platelet count are required in patients treated with volanesorsen. Recently, hepatic steatosis was shown to be reduced with volanesorsen in patients with severe hypertriglyceridaemia, familial partial lipodystrophy and familial chylomicronaemia syndrome (FCS) [112]. In May 2019, volanesorsen was approved by the EMA for the treatment of adult patients with FCS. This was based on the positive and beneficial results of the multinational phase 3 APPROACH and COMPASS trials, which were conducted despite adverse effects. However, the FDA has denied approval for volanesorsen. There is another antisense oligonucleotide that targets Apo-CIII and has a different conjugation (N-acetyl-galactosamine): AKCEA-APOCIII -Lrx or ISIS 678354. It has been studied in a phase 1/2a trial in single-dose and multiple-dose regimens. In cohorts treated with single doses of 10, 30, 60, 90 or 120 mg AKCEA-APOCIII -LRx, reductions of −12%, −7%, −42%, −73% and −77% were achieved in TG levels [113]. In cohorts with multiple doses of 15 and 30 mg weekly and 60 mg every 4 weeks, −59%, −73% and −66% reductions in TGs were observed 1 week after the last dose [113]. This compound has not been associated with a reduction in platelet count or flu-like symptoms, so this new ASO has the potential to replace volanesorsen in patients with FCS [113]. Olezarsen is another antisense oligonucleotide that inhibits Apo-CIII. It is currently in phase 3 clinical trials. A phase 2 clinical trial investigated four different treatment regimens and reported mean percentage TG reductions of 23% with 10 mg every 4 weeks, 56% with 15 mg every 2 weeks, 60% with 10 mg every week and 60% with 50 mg every 4 weeks, compared with an increase of 6% for the pooled placebo group 6 months after starting therapy [114]. In another phase 2 study, a 51% decrease in TGs and a 15% increase in HDL-cholesterol were seen in association with olezarsen treatment [115]. It appears that olezarsen has a better safety profile than volanesorsen, as thrombocytopenia and injection site reactions were the only adverse effects reported [114]. An siRNA therapy against Apo-CIII is also being investigated—ARO-APOC31001. It has been studied in a phase 1 trial in healthy volunteers, patients with hypertriglyceridaemia and patients with FCS. A 41–55% TG reduction without severe side effects was reported [104]. More studies are ongoing, but the preliminary data for these new drugs are promising.

### 4.4. Mipomersen

Apolipoprotein B is an important structural protein of VLDL, LDL and Lp(a). It has already been mentioned that cholesterol-rich lipoprotein particles are the main factor for increased cardiovascular risk. The production of apolipoprotein B has been targeted by new drugs such as mipomersen—an antisense oligonucleotide that inhibits apolipoprotein B production. Early studies with mipomersen showed that it produced good changes in lipid profiles which led to its approval for the treatment of HoFH [116]. Mipomersen is administered subcutaneously once weekly at a dose of 200 mg. A meta-analysis of the efficacy of mipomersen found that it significantly lowered LDL-cholesterol (weighted mean difference (WMD) −1.55), non-HDL-cholesterol (WMD −1.66), Lp(a (WMD −0.99), TG (WMD −0.61) and apolipoprotein B (WMD −1.66) [104]. However, this meta-analysis also reported significant adverse effects, such as injection site reactions, flu-like symptoms, liver toxicity and hepatic steatosis [104,117]. Liver toxicity and hepatic steatosis, as well as concerns about potential long-term safety, led to its rejection by the EMA in 2012 and again in 2013, and to the withdrawal of mipomersen from the market in 2019. Currently, there are no further studies with mipomersen or similar drugs, as it is believed that the silencing of the apolipoprotein B gene has a toxic effect on the liver [104].

## 5. Future Possibilities

### 5.1. Lomitapide

Lomitapide is an inhibitor of microsomal triglyceride transfer protein (MTP), a protein responsible for the transfer of triglycerides into apolipoprotein B-rich particles and the formation of VLDL [9]. Inhibition of MTP causes a decrease in apolipoprotein B secretion in the liver and decreases serum LDL-cholesterol levels. In patients with HoFH, the treatment with this drug is associated with an up to 60% reduction in LDL-cholesterol [118]. Frequently reported adverse effects include gastrointestinal discomfort (up to 40%), elevation of liver enzymes, and hepatic steatosis with or without concomitant increases in transaminases but there was no sign of increased risk of liver fibrosis [118]. It also induces autophagic cell death via inhibition of mTOR and could therefore be potentially used in the future as a new option for cancer treatment [119]. However, further studies have to be conducted in order to assess lomitapide’s potential in this new indication. It is administered orally at a dose of 5-10 mg and it is approved by the EMA and FDA for the treatment of patients with HoFH.

### 5.2. Lerodalcibep

Lerodalcibep is a drug that inhibits PCSK9 via the CRISPR–Cas9 (Clustered Regularly Interspaced Short Palindromic Repeats) technique. CRISPR–Cas9 is a gene-editing technique that utilises a guide RNA and the Cas9 protein to modify specific DNA sequences within an organism’s genome with high precision. Lerodalcibep is a recombinant fusion protein of a PCSK9-binding domain (adnectin) and human serum albumin that increases its half-life to 12–15 days, enabling once a month administration [120]. It has progressed to phase 3 clinical trials with excellent unofficial media-published results from the phase 2 trials: 77.3% LDL-cholesterol reduction at 12 weeks with 300 mg every 4 weeks and 33.5% LDL-cholesterol reduction at week 12 with 150 mg every 4 weeks. Subsequent studies confirmed this data and reported a stable 60% mean LDL-cholesterol reduction [121]. Currently there are six phase 3 trials analysing the effect of this drug in patients with HoFH and atherosclerotic CVD, and studies of primary prevention in high-risk patients with lerodalcibep are ongoing.

### 5.3. PCSK9 Vaccines

A newly emerging strategy is vaccines that would cause intrinsic production of antibodies against PCSK9. A novel anti-PCSK9 vaccine formulation, called liposomal immunogenic fused PCSK9–tetanus peptide plus alum adjuvant (L-IFPTA), was recently designed [121]. It induces long-lasting PCSK9 antibody production and reduced LDL-cholesterol by 51.7% in BALB/c mice and by 19.2% in C57BL/6 mice [121]. Although it is relatively new, this field is promising because it would possibly not require frequent administrations like some previously mentioned therapy options. 

## 6. Discussion

As mentioned in the introduction, dyslipidaemia is an important risk factor for atherosclerotic CVD. It is considered the most important modifiable risk factor. It can be improved by dietary changes, physical activity and various medications that affect the lipid profile. The European Society of Cardiology and the European Atherosclerosis Society recommend statins as the first line of treatment for dyslipidaemia. They base this on studies that have shown an up to a 23% reduction in severe coronary events and a 10% reduction in all-cause mortality over 5 years [9,12]. Such a reduction in adverse effects is only reported for statins. The studies on the other agents discussed in this paper have so far focused only on the effects of lowering LDL-cholesterol or TGs. There are currently several studies analysing the cardiovascular consequences of using different lipid-lowering agents, and preliminary data are expected in the near future. The main obstacle to achieving the targeted lipid levels with statin therapy is low adherence to statin therapy. Although statin therapy is rarely associated with serious adverse effects, it is still burdened in the public perception by the high risk of muscle and liver damage. The occurrence of statin intolerance has been analysed in placebo-controlled trials. The data show that actual intolerance is not nearly as common as reported and that adults who have proven statin intolerance could be prescribed other approved lipid-lowering agents [19,122]. The risk of uncontrolled dyslipidaemia far outweighs the risk of serious adverse effects associated with statin therapy. Therefore, patients should be thoroughly educated and lipid lowering should be the focus of primary health care in patients at risk of CVD. LDL-cholesterol target levels are trending downwards. Current guidelines recommend LDL-cholesterol levels of <1.4 mmol/L for both primary and secondary prevention in patients at very high risk, with LDL-cholesterol targets trending even lower in the future. To achieve such low LDL-cholesterol levels and further reduce the risk of cardiovascular outcomes, add-on therapies with new drugs should be initiated in patients already receiving statin therapy. Newer drugs should be an option for patients with proven statin intolerance. The addition of ezetimibe and PCSK9 to statins is already associated with an additional reduction in cardiovascular risk. Further outcome studies for some of the drugs discussed in this article will provide the data needed to adjust guidelines for maximum cardiovascular disease risk reduction, especially in patients at very high and extremely high CVD risk.

## 7. Conclusions

Lipid-lowering drugs are already among the most commonly prescribed medications, with atorvastatin expected to be the most prescribed drug in the US by 2020 [123]. As dyslipidaemia remains an important cardiovascular risk factor, the development of new lipid-lowering drugs is progressing rapidly. Some agents such as PCSK9 inhibitors, inclisiran and ANGPTL3 inhibitors are already approved drugs that show great potential and promise wider use and lower costs in the near future. Although statins are among the most commonly prescribed drugs worldwide and new drugs are entering the market, dyslipidaemia is still often uncontrolled and represents a major burden for the healthcare system. A change in approach from focusing strictly on LDL-cholesterol levels to a more comprehensive analysis of the lipid profile is something we could expect in the future, as other lipoprotein particles also contribute to residual cardiovascular risk. Given the good results of statin therapy in lowering lipid levels, its use should be encouraged, with careful consideration of discontinuation. True statin intolerance is not common and the risk/benefit ratio of discontinuing therapy is unfavourable, especially in high-risk and very-high-risk patients. In the future, it is expected that combination therapy will become more common compared to statin monotherapy, with new agents being used in addition to statin therapy. Outcome and safety profile studies already underway are evaluating the endpoints of the new treatments and will provide insight into the potential benefits of these agents. With the current data reporting excellent outcomes, there are strong indications of more successful reductions in MACEs in the years ahead.

## Figures and Tables

**Table 1:** Overview of lipid-lowering agents focusing on lipid profile, CVE outcomes and side effects.

Drug	Mechanism of Action	Lipoprotein Affected	CVE Outcomes	Indication	Side Effects
**Statins**	HMG-3-CoA reductase antagonists	Total-C LDL-C Apo(B) TG	All-cause mortality reduction (OR 0.86, 95%CI 0.79 to 0.94)	Primary and secondary dyslipidaemia Prevention of CVD	Muscle pain Liver injury Insulin resistance
**Bempedoic acid**	ATP citrate lyase inhibitor	LDL-C Apo(B) nHDL-C	LDL-C reduction (−17.4 %, −18.1%, −21.4%) compared to placebo at 12 weeks All-cause mortality reduction (HR 0.73 95%CI 0.54–0.98)	Primary hypercholesterolaemia Mixed dyslipidaemia	Gout Cholelithiasis Elevated serum creatinine, uric acid and hepatic enzymes
**Ezetemibe**	NPC1L1 Transporter inhibitor	Total-C LDL-C TG	LDL-C reduction(−18.58%) Total-C reduction (−13.46%) TG reduction (−8.06%)	Primary hypercholesterolaemia Mixed dyslipidaemia	Abdominal pain Diarrhoea Flatulence
**Evolocumab Alirocumab**	PCSK9 inhibitors—monoclonal antibodies	LDL-C Apo(B) nHDL-C Lp(a)	LDL-C reduction (−50–60% non-HoFH, −30% HoFH) Lp(a) reduction (−38.84%) MACE reduction (HR 0.85, 95%CI 0.79–0.92, *p* < 0.001)	Primary hypercholesterolaemia Mixed dyslipidaemia HeFH HoFH Prevention of CVD	Administration site reaction Flu-like symptoms Vitamin E levels reduction
**Inclisiran**	PCSK9 mRNA translation interfering—siRNA	LDL-C Apo(B) nHDL-C Lp(a) TG	LDL-C reduction (−50%)	Primary hypercholesterolaemia Mixed dyslipidaemia HeFH HoFH Prevention of CVD	Administration site reaction Flu-like symptoms
**Evinacumab**	ANGPTL3 inhibitor—monoclonal antibody	LDL-C Apo(B) nHDL-C TG	LDL-C reduction (−49%,−59%) TG reduction (−77%, −83%)	HoFH	Flu-like symptoms Elevated serum liver enzymes Hepatic steatosis (Vupanorsen discontinued)
**Pelacarsen**	Blocking Apo(A) translation—ASO	LDL-C Lp(a) Apo(B)	Lp(a) reduction (−29% to −67%) LDL-C reduction (−2% to −19%)	Not approved	Myalgia UTIs Administration site reaction Headache
**Olpasiran**	Lp(a) mRNA translation inhibitor—siRNA	Lp(a)	Lp(a) reduction (<90%)	Not approved	Not assessed
**Volanesorsen**	ApoC-III mRNA translation interefering—ASO	ApoC-III TG Chylomicrons	TG reduction (−74%) ApoC-III reduction (−80%)	Hypertriglyceridaemia Hyperchylomicronaemia FCS	Administration site reaction Upper respiratory tract infections Thrombocytopenia Serum sickness
**Olezarsen**	ApoC-III mRNA translation interefering—ASO	ApoC-III TG Chylomicrons	TG reduction (−51%)	Not approved	Administration site reaction

Abbreviations: HMG-3-CoA (3-hydroxy-3-methylglutaryl coenzyme A), Total-C (Total Cholesterol), LDL-C (LDL Cholesterol), Apo(B) (Apolipoprotein (B)), TG (Triglycerides), OR (Odds ratio), CVD (Cardiovascular disease), nHDL-C (non-HDL cholesterol), HR (Hazard ratio), NPC1L1 (Neimann-Pick C1 like 1), PCSK9 (Proprotein convertase subtilisin/kexin type 9), Lp(a) (Lipoprotein (a)), HoFH (Homozygous familial hypercholesterolaemia), HeFH (heterozygous familial hypercholesterolaemia), MACE (Major cardiovascular event), siRNA (Small interfering RNA), ANGPTL3 (Angiopoetin-like 3 protein), Apo(A) (Apolipoprotein (A)), ASO (Antisense oligonucleotide), ApoC-III (Apolipoprotein C-III), FCS (Familial chylomicronaemia syndrome).

## Data Availability

Not applicable.
